# Consumption

**Published:** 1860

**Authors:** J. G. Westmoreland

**Affiliations:** Atlanta, Ga.


### ARTICLE III.

*Consumption.* By J. G. Westmoreland, M. D., Atlanta, Ga.

The name which heads this article has been applied to a peculiar constitutional disease, affecting, generally, the lungs in a manner leading finally to a destruction, or *consuming* of the substance of a portion of those organs. The same amount of destruction of the tissues may occur from other causes, producing the same general and physical symptoms, to which this term does not properly belong. Valid objections are made to the term *consumption.* It has no specific meaning by which the true character of the disease may be inferred, nor even the particular organ that may be consumed or destroyed.

This constitutional derangement, leading so generally to consumption of the substance of the lungs, does not always affect these organs, even when running on to a fatal termination. The bowels, brain and other importantviscera and tissues are subject to destruction from the same cause. Hence, the general Term Tuberculosis is given to that derangement of the system in which we find that disease developed, called *consumption.* And not only so, but when the pulmonary organs are the subject specially of the destructive process, the distinctive appellation Tubercular Phthisis, or Phthisis Pulmonalis, is used to give, not only a a name to the constitutional disturbance, but the special organic lesion that exists.

As above intimated, destruction of the pulmonary structures may occur without Tuberculosis. As a result of ordinary inflammations of the parenchyma, either chronic or acute, the ulcerative process may be established, and so far as the effect upon the economy is concerned, not only produce the same symptoms but exhibit the same local pathological condition after the breaking down of the tissues; for in genuine Tubercular Phthisis, as will be hereafter noticed more particularly, inflammation and irritation precedes the ulcerative process, and is caused by the Tubercular deposit acting as a foreign substance. A traumatic injury of any kind, received in the substance of the lungs, may lead to the same pathological condition, and to the same characteristic symptoms of Tubercular Consumption ; and the difference observed in the progress and termination, from these different causes, is attributable to the continuance of the irritating deposite in Tuberculosis, increasing the ulcerative process where it exists, and involving new portions.

Then, the expressive terms, Tuberculosis and Tubercular Phthisis, convey more definitely the idea intended generally by Consumption, the former, when such disease exists in the respiratory organs or without involving the lungs, the latter when those organs give evidence of tubercular deposite in them.

"There is something in a name" to those who wish, in naming, to express something practical, and in learning terms, to be able to associate with them something more than arbitrary, empty sound.

[TO BE CONTINUED.]

